# Transient bilateral chorea secondary to digoxin toxicity in a female with acute kidney injury: a case report

**DOI:** 10.1093/ehjcr/ytab022

**Published:** 2021-02-04

**Authors:** James Mannion, Samreen Tariq, Patrick Owens

**Affiliations:** Cardiology Department, University Hospital Waterford, Waterford, Ireland

**Keywords:** Digoxin toxicity, Acute kidney injury, Chorea, Movement disorder, Case report

## Abstract

**Background:**

Chorea secondary to digoxin toxicity is rare, with only three published cases describing the phenomenon. We report the case of a 78-year-old female presenting with intermittent vomiting and diarrhoea for 4 weeks. She had a history of chronic kidney disease and digoxin use for atrial fibrillation.

**Case summary:**

A 78-year-old lady presented to the emergency department with a 4-week history of intermittent vomiting and diarrhoea. These symptoms commenced after a course of antibiotics prescribed by her general practitioner for a urinary tract infection. Her admission electrocardiogram demonstrated atrial fibrillation at a rate of 32, with evidence of digitalis toxicity. Her creatinine was 396 µmol/L (44–80 µmol/L) with digoxin level 8.1 nmol/L (0.77–1.5 nmol/L). Initially, treatment was with digoxin-specific antibody (FAB) and fluid resuscitation. Within 24 h, she developed transient head, neck, and bilateral upper limb chorea. Review of medications revealed no other likely causative agent. Neuroimaging showed no new ischaemia, but stable established bilateral infarcts of the basal ganglia. Haloperidol 0.5 mg twice daily was commenced. Three days later as digoxin levels normalized, the chorea resolved entirely without recurrence.

**Discussion:**

We have identified three reported cases of digoxin-induced chorea. Our case resembles two of the published cases where a transient bilateral chorea, associated with digitalis toxicity and resolving within a few days of normalization of digoxin levels was demonstrated. There were no other focal neurological signs or symptoms. It has been postulated that an alteration to dopaminergic neuronal activity is a potential mechanism, as digoxin also demonstrates neuropsychiatric side effects such as psychosis and depression.

**Learning points**

- Chorea is a rare complication of digoxin toxicity.
- This condition is managed acutely with dopamine blocking agents, but mainstay of treatment is reversal of digoxin and supportive care.
- Those with prior established infarct of the basal ganglia may be more likely to develop this complication.

## Introduction

Chorea is a rare movement disorder characterized by its involuntary, rapid, and irregular movements. It sometimes resembles purposeful motion and can be described as ‘dance-like’, hence is derived from the Ancient Greek word meaning ‘dance’. Although rare, causes of this phenomenon are extensive, including vascular, endocrine, drug-related, inflammatory, or genetic causes. In patients with atrial fibrillation, this phenomenon is commonly secondary to cardio-embolic stroke.^1^

Digoxin has a narrow therapeutic window, and serum toxicity has well-recognized effects on the central nervous system such as encephalopathy, hallucinations, seizures, dysphagia, dysphonia, and visual disturbances.^2^ Chorea, however, is an exceedingly rare side effect of digoxin toxicity, described only three times in the literature since 1984.^3–5^ The typical presentation occurs as a transient bilateral chorea, in the setting of digitalis toxicity, which resolves within a few days of normalization of digoxin levels and is associated with no other focal neurological signs or symptoms.

## Timeline

**Table:** 

Timeline	Admission	Within 24 h	72 h	1 month
Movements	Normal	Bilateral chorea	Chorea resolved	Normal
Digoxin level	Toxic	Toxic	Normalized	Bisoprolol used as an alternative.
Renal function	Acute kidney injury (AKI) on chronic kidney disease (CKD)	AKI on CKD	Baseline CKD	Baseline CKD

## Case presentation

A 78-year-old lady presented to the emergency department with a 4-week history of intermittent diarrhoea and vomiting in addition to fatigue and malaise. This began after she completed a course of antibiotics, which were prescribed by her general practitioner to treat a urinary tract infection.

She was an ex-smoker and had a past medical history of atrial fibrillation (AF), chronic kidney disease, chronic obstructive pulmonary disease, gout, type-2 diabetes mellitus.

On admission, our patient was regularly taking edoxaban 30 mg once daily (o.d.), digoxin 125 micrograms (mcg) o.d., bisoprolol 5 mg o.d., amiodarone 200 mg o.d., allopurinol 100 mg o.d., ferrous fumarate 305 mg o.d., lansoprazole 30 mg o.d., metformin 500 mg three times daily, gliclazide (modified release) 30 mg o.d., furosemide 40 mg o.d., atorvastatin 20 mg o.d., budesonide/formoterol 200 mcg/6 mcg inhaled twice daily, umeclidinium 55 mcg inhaled o.d., mirtazapine 30 mg o.d., in addition to nutritional supplementation.

Observations on arrival found a heart rate of 40 beats per minute, with a blood pressure of 103/57. Respiratory rate was 24 with an oxygen saturation of 92% on room air. She was apyrexial. She had cool peripheries with an otherwise normal examination.

**Figure 1:**
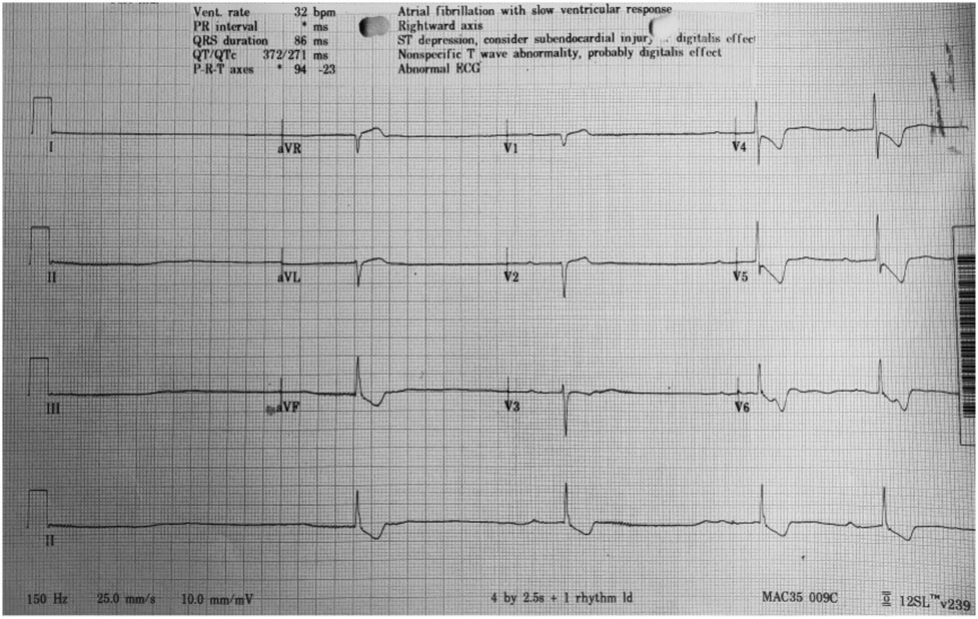
Electrocardiogram of patient on admission to the emergency department. This demonstrates slow atrial fibrillation with diffuse down-sloping ST segments in-keeping with digoxin toxicity.

Serial electrocardiograms demonstrated bradycardic AF (see *Figure 1*), with a ventricular rate between 32 and 52 b.p.m. T-wave inversion with down-sloping ST depression was present in leads II, III, aVF, and V3–V6. Serology showed an acute kidney injury (AKI) with hyperkalaemia [creatinine 396 µmol/L (44–80 µmol/L), eGFR 10 mL/min/1.73 m^2^ and potassium 6.2 mmol/L]. Digoxin levels were markedly elevated at 8.1 nmol/L (0.8–1.2).

C-reactive protein was 3.8 mg/L (0–5). Her liver function tests showed total bilirubin 4.9 µmol/L (2.0–21), alkaline phosphatase 99 IU/L (30–130), gamma GT 114 U/L (6–42), and albumin 42 g/L (35–50).

Other bloods included venous blood gas: pH 7.42 (7.35–7.45), lactate 4.4 mmol/L (0.0–1.3), glucose 5.2 mmol/L (3.6–5.3). Full blood count: haemoglobin 12.2 (12–15), white cell count 9.9 × 10^9^/L (4–10), platelets 263 × 10^9^/L (150–400). Electrolyte screen: sodium 144 mmol/L (135–145), magnesium 0.57 mmol/L (0.7–1.0), calcium 2.59 mmol/L (2.2–2.6), phosphorus 1.1 mmol/L (0.8–1.5).

Echocardiography was done the following day and demonstrated no regional wall motion abnormality, with good ejection fraction (50%) and normal valves

In terms of medications administered, in the first 24 h of her admission, our patient received management for hyperkalaemia [i.e. 10 mL of 10% calcium gluconate intravenously (IV), with 10 units actrapid in 50 mL of 50% dextrose IV infusion], five vials of digoxin FAB, resuscitation with NaCl 0.9% IV, magnesium sulfate 2 g IV, and an IV dobutamine infusion.

She was administered with intravenous digoxin specific antibody (FAB), treated for hyperkalaemia (6.2 mmol/L), commenced on fluid resuscitation, and transferred to the coronary care unit (CCU) for cardiac monitoring. While in the CCU, she was noted to develop generalized symmetrical choreiform movements. She had not been treated with any neuroleptic medications or any other drugs. Examination revealed myoclonus in the fingers and dyskinetic movements of the oral muscles and tongue. Diffuse hypertonia and hyper-reflexia were present. An urgent computerized tomography brain scan showed no new abnormalities but demonstrated old established basal ganglia infarcts bilaterally.

Magnetic resonance imaging would be the preferred method of investigation in this setting; however, this was not acutely available in our centre.

She was reviewed by neurology and commenced on 0.5 mg of haloperidol twice daily. Her clinical state improved in correlation with supportive measures for haemodynamics and renal function.

Symptoms of chorea resolved fully as digoxin levels normalized without recurrence even after cessation of haloperidol, and the patient was discharged home after 5 days. The absence of chorea was confirmed at a virtual clinic 1 month after her discharge.

## Discussion

We have identified three reported cases of digoxin-induced chorea, published since 1984.^3–5^ Two cases involved bilateral choreiform movements, in the setting of acute toxicity—one case secondary to reduced clearance following AKI, as with our patient. The movements were transient, resolving within a few days of treatment of the toxicity. Typical antipsychotics such as haloperidol were used with good effect in the acute setting, and successful weaning occurred if digoxin levels were no longer elevated. When one case was revisited 2 years later, no recurrence of chorea had occurred.^4^

A second case involved a 7-year-old girl with congenital heart disease, who was initiated on 125 micrograms of digoxin twice daily, and developed choreiform movements when her digoxin level rose to 3.8 ng/mL. This disappeared when levels fell to 1.5 ng/mL and recurred when she was re-trialled on the drug with levels rising to 2.5 ng/mL. Symptoms fully resolved when digoxin was discontinued and blood levels fell back to the normal range.^5^

The final case was unique to the others as hemi-chorea was noted. The author described a 76-year-old lady who newly commenced digoxin in the setting of new AF and heart failure. Despite ceasing digoxin, the chorea did not fully resolve, even after 1 week. The author adds that acute ischaemic stroke could not be excluded as the cause.^3^

Our case resembles two of the published cases where a transient bilateral chorea, associated with digitalis toxicity, resolves within a few days of normalization of digoxin levels and is associated with no other focal neurological signs or symptoms. As digoxin is used for rate control in AF, patients are at an increased risk of cardio-embolic stroke, and this is a leading differential diagnosis if the symptoms are unilateral or the symptoms do not fully resolve soon after resolution of toxicity.^6^

Of note, amiodarone has been demonstrated to cause neuromuscular side effects such as ataxia and generalized hyper-reflexia when at toxic levels. This, however, has been described as taking 2–6 months to resolve, and does not clinically fit the choreiform movements at rest exhibited by our patient.^7^^,^^8^

All of the patient’s regular medications except for inhalers, anticoagulation, and nutritional supplements were held in the first 24 h. Although mirtazapine is known to cause psychomotor agitation and other forms of hyperkinesia when reduced clearance is present, there are no published reports of mirtazapine toxicity causing chorea.^9^^,^^10^ There were also no identifiable agents that are known to induce chorea upon their withdrawal.

The cause of digoxin-related chorea is not known, however, it has been postulated that an alteration to dopaminergic neuronal activity is a potential mechanism as digoxin also demonstrates neuropsychiatric side effects such as psychosis and depression.^11^ As demonstrated with our patient’s neuroimaging, there were old bilateral infarcts of the basal ganglia. This established parenchymal loss may have acted as a predisposing factor to developing chorea in the setting of digoxin toxicity.

Due to the narrow therapeutic window, digoxin therapy needs to be monitored more strictly in those who are at risk of digitalis toxicity, such as in the setting of chronic kidney disease. It is also important to fully educate patients to be vigilant of the side effects of digoxin and to present themselves to a healthcare professional in a timely manner to avoid haemodynamic compromise.

## Lead author biography

**Figure:**
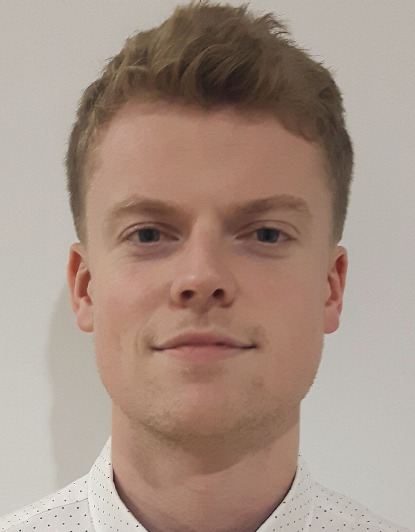


Dr James Mannion, MB BCh BAO, is a cardiology Senior House Officer, working in University Hospital Waterford, in the south-east of Ireland. He is currently enjoying his second year of his Basic Specialist Training programme and hopes to go onto Higher Specialist Training in cardiology and thereafter electrophysiology.

## Supplementary material

Supplementary material is available at *European Heart Journal - Case Reports* online.

**Slide sets:** A fully edited slide set detailing these cases and suitable for local presentation is available online as Supplementary data.

**Consent:** The authors confirm that written consent for submission and publication of this case report including images and associated text has been obtained from the patient in line with COPE guidelines.

**Conflict of interest:** None declared.

**Funding:** None declared.

## Supplementary Material

ytab022_Supplementary_Data
