# Supplementary material for: Transient bilateral chorea secondary to digoxin toxicity in a female with acute kidney injury: a case report
Source: Eur Heart J Case Rep. 2021 Feb 4;5(2):ytab022. doi: 10.1093/ehjcr/ytab022 (PMC7859595; doi:10.1093/ehjcr/ytab022)
Supplement: ytab022_Supplementary_Data [file ytab022_supplementary_data.pptx]

## Slide 1
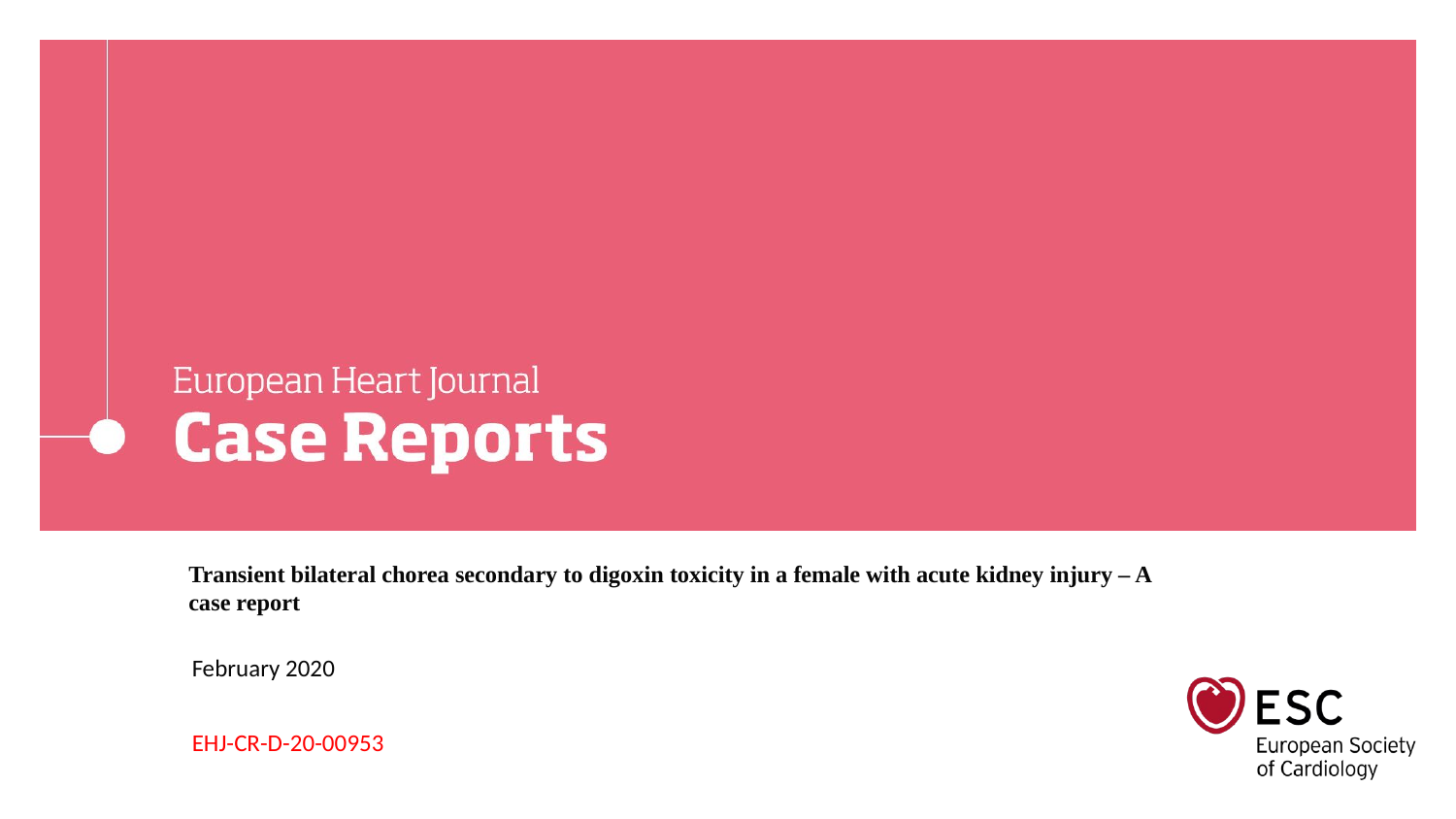

# Transient bilateral chorea secondary to digoxin toxicity in a female with acute kidney injury – A case report
February 2020
EHJ-CR-D-20-00953

## Slide 2
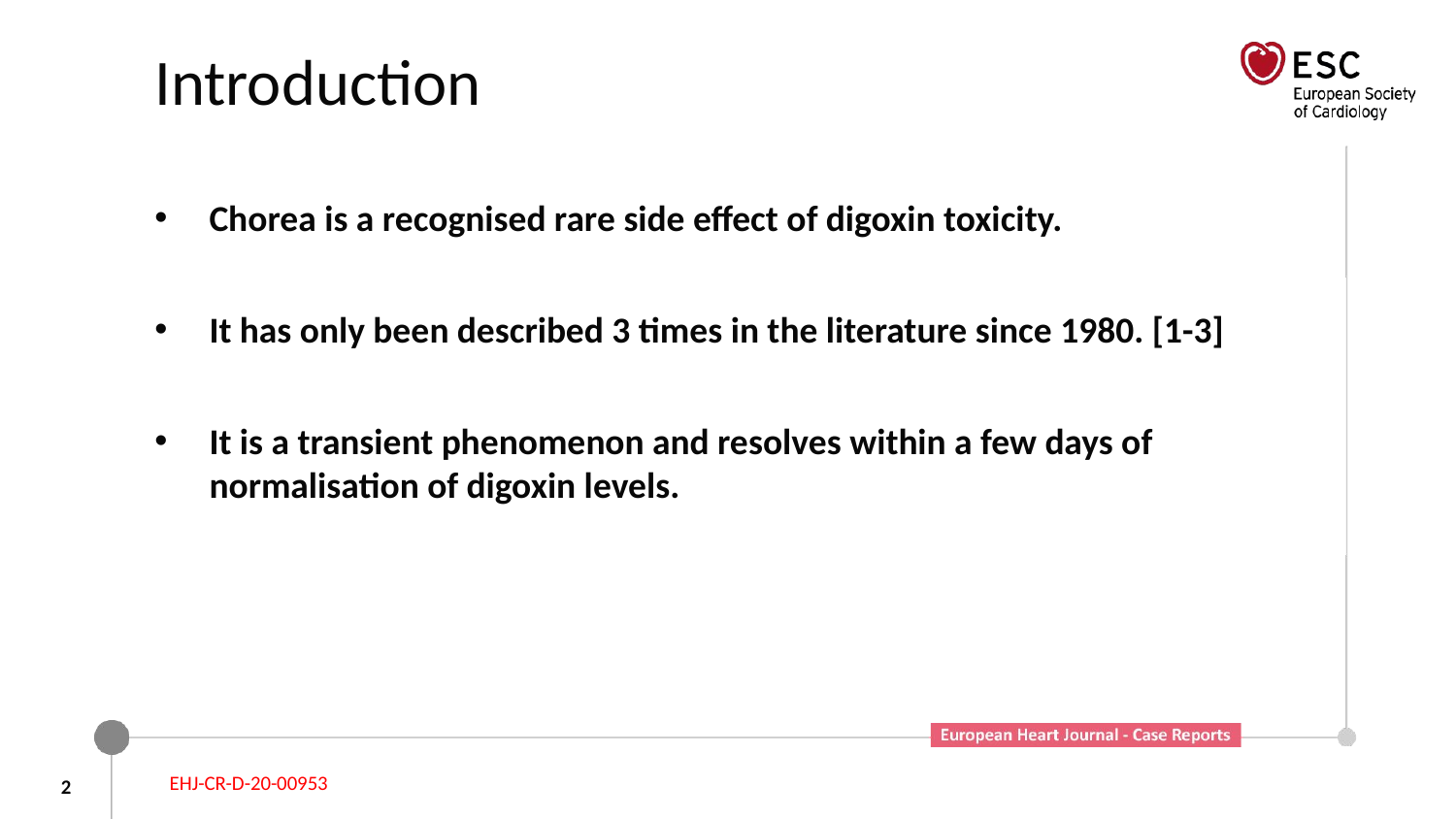

# Introduction
Chorea is a recognised rare side effect of digoxin toxicity.
It has only been described 3 times in the literature since 1980. [1-3]
It is a transient phenomenon and resolves within a few days of normalisation of digoxin levels.
2
EHJ-CR-D-20-00953

## Slide 3
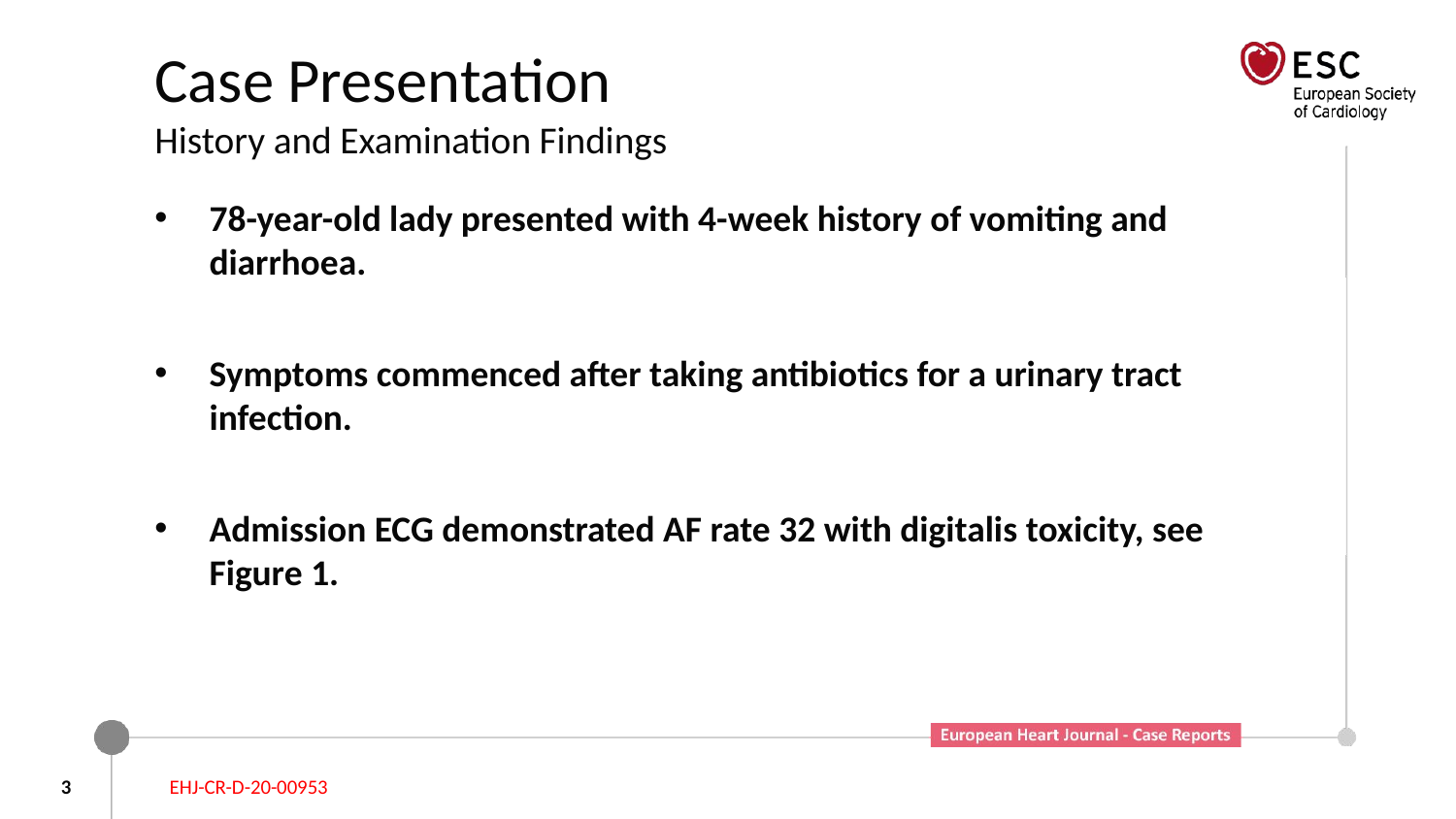

# Case Presentation History and Examination Findings
78-year-old lady presented with 4-week history of vomiting and diarrhoea.
Symptoms commenced after taking antibiotics for a urinary tract infection.
Admission ECG demonstrated AF rate 32 with digitalis toxicity, see Figure 1.
3
EHJ-CR-D-20-00953

## Slide 4
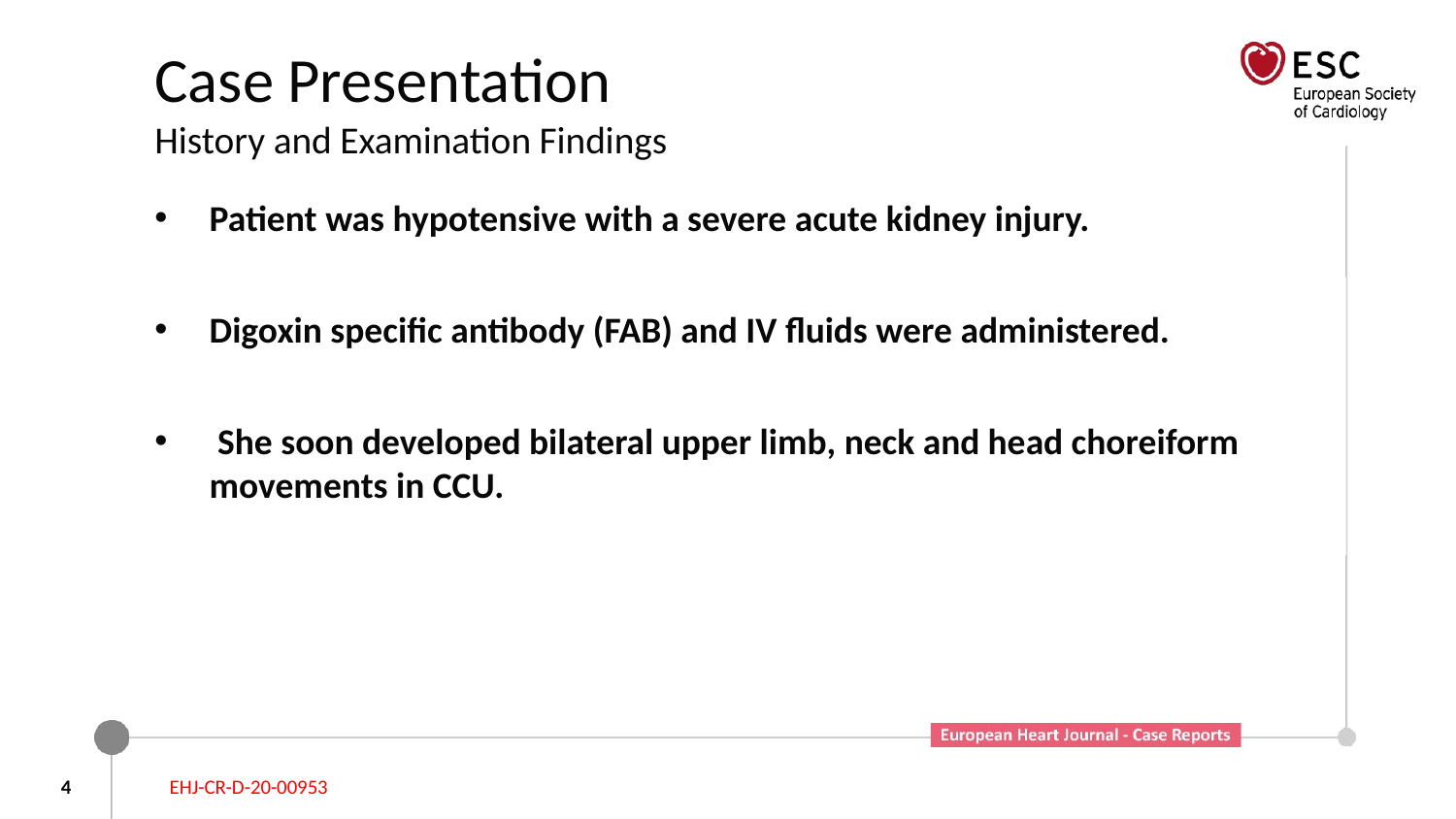

# Case Presentation History and Examination Findings
Patient was hypotensive with a severe acute kidney injury.
Digoxin specific antibody (FAB) and IV fluids were administered.
 She soon developed bilateral upper limb, neck and head choreiform movements in CCU.
4
EHJ-CR-D-20-00953

## Slide 5
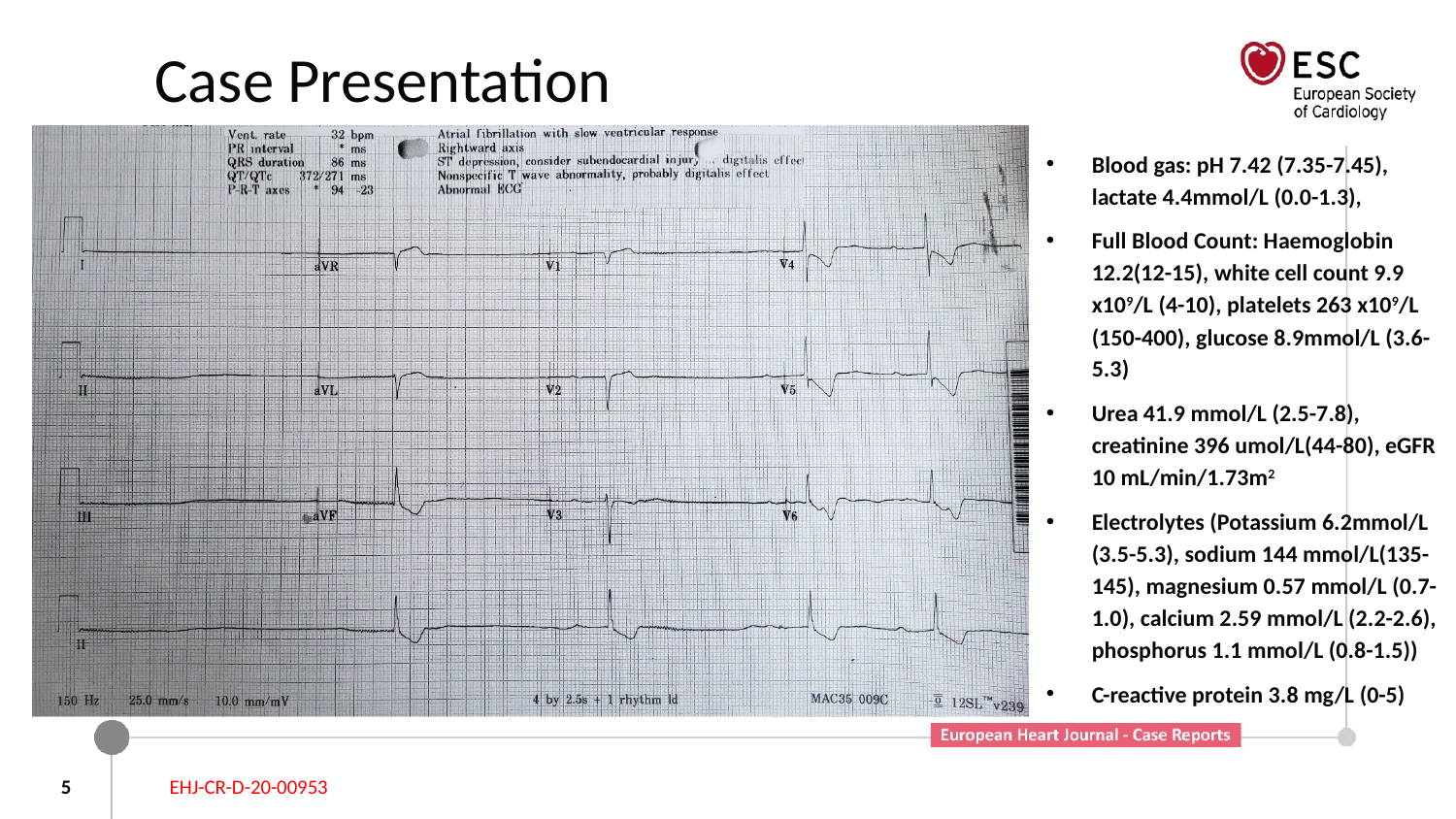

# Case Presentation Investigations: Bloods and ECG
Blood gas: pH 7.42 (7.35-7.45), lactate 4.4mmol/L (0.0-1.3),
Full Blood Count: Haemoglobin 12.2(12-15), white cell count 9.9 x10^9^/L (4-10), platelets 263 x109/L (150-400), glucose 8.9mmol/L (3.6-5.3)
Urea 41.9 mmol/L (2.5-7.8), creatinine 396 umol/L(44-80), eGFR 10 mL/min/1.73m2
Electrolytes (Potassium 6.2mmol/L (3.5-5.3), sodium 144 mmol/L(135-145), magnesium 0.57 mmol/L (0.7-1.0), calcium 2.59 mmol/L (2.2-2.6), phosphorus 1.1 mmol/L (0.8-1.5))
C-reactive protein 3.8 mg/L (0-5)
Figure 1:
Figure 1: Admission ECG demonstrating diffuse ST segment depression and bradycardia.
5
EHJ-CR-D-20-00953

## Slide 6
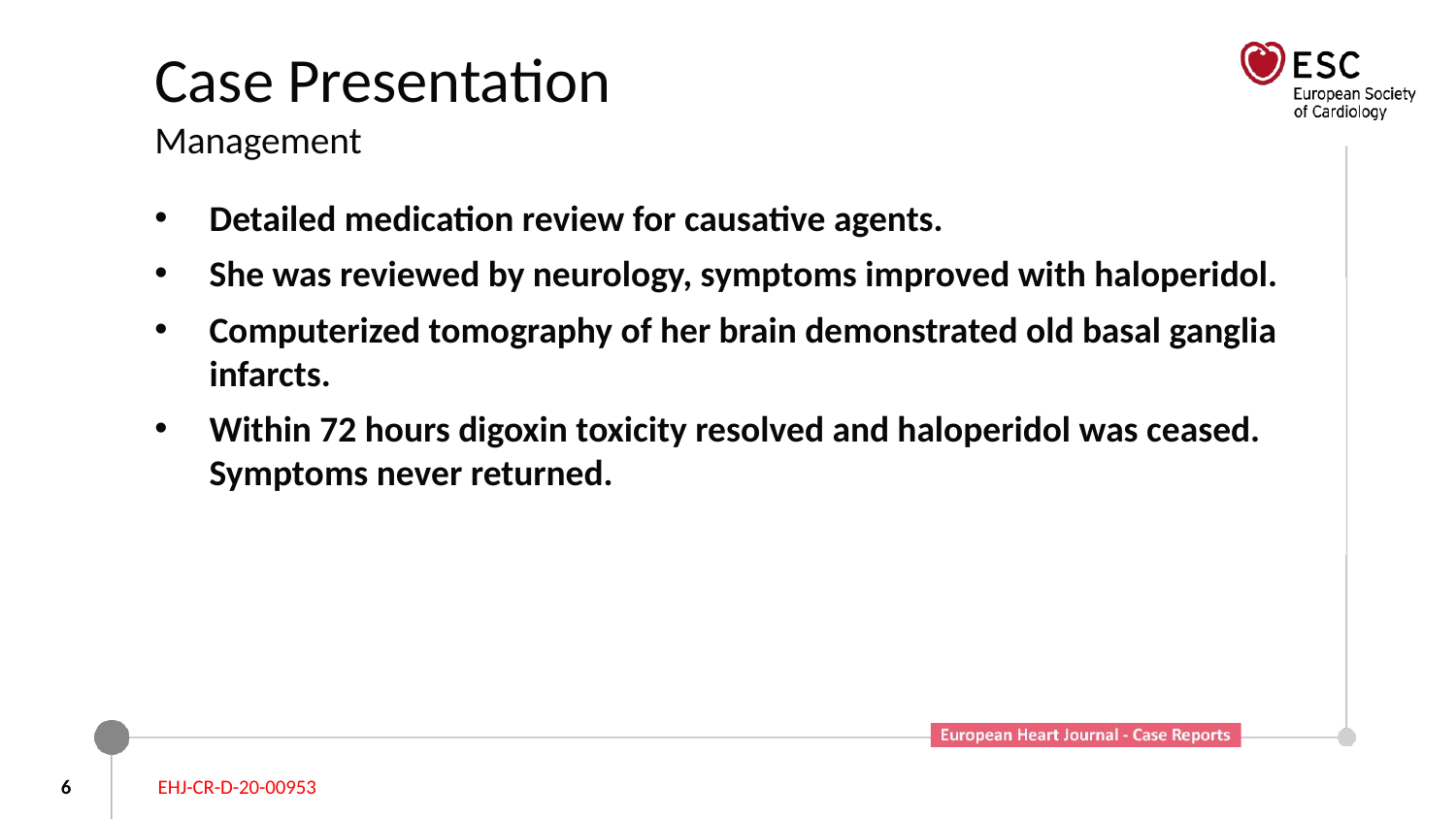

# Case Presentation Management
Detailed medication review for causative agents.
She was reviewed by neurology, symptoms improved with haloperidol.
Computerized tomography of her brain demonstrated old basal ganglia infarcts.
Within 72 hours digoxin toxicity resolved and haloperidol was ceased. Symptoms never returned.
6
EHJ-CR-D-20-00953

## Slide 7
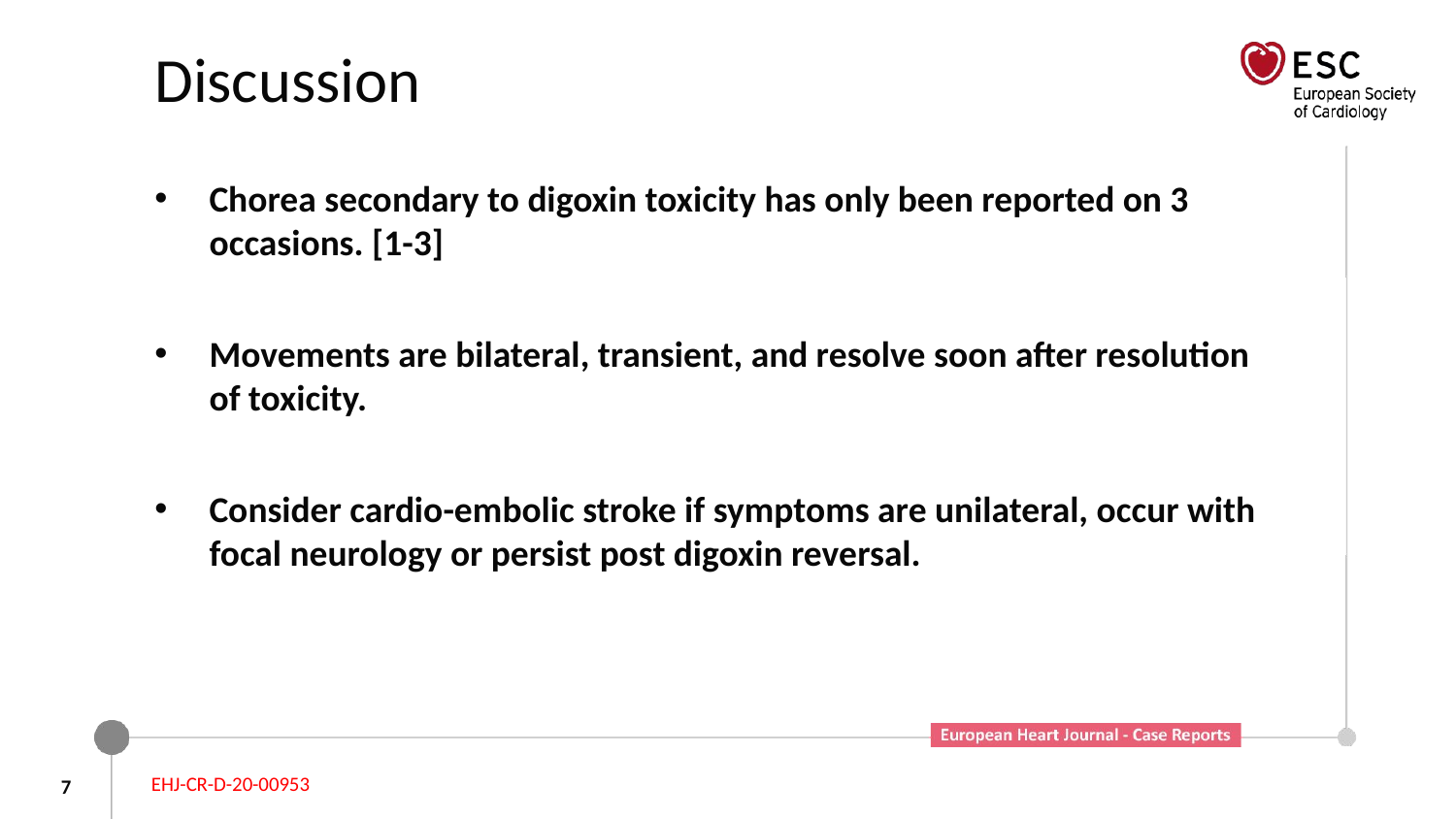

# Discussion
Chorea secondary to digoxin toxicity has only been reported on 3 occasions. [1-3]
Movements are bilateral, transient, and resolve soon after resolution of toxicity.
Consider cardio-embolic stroke if symptoms are unilateral, occur with focal neurology or persist post digoxin reversal.
7
EHJ-CR-D-20-00953

## Slide 8
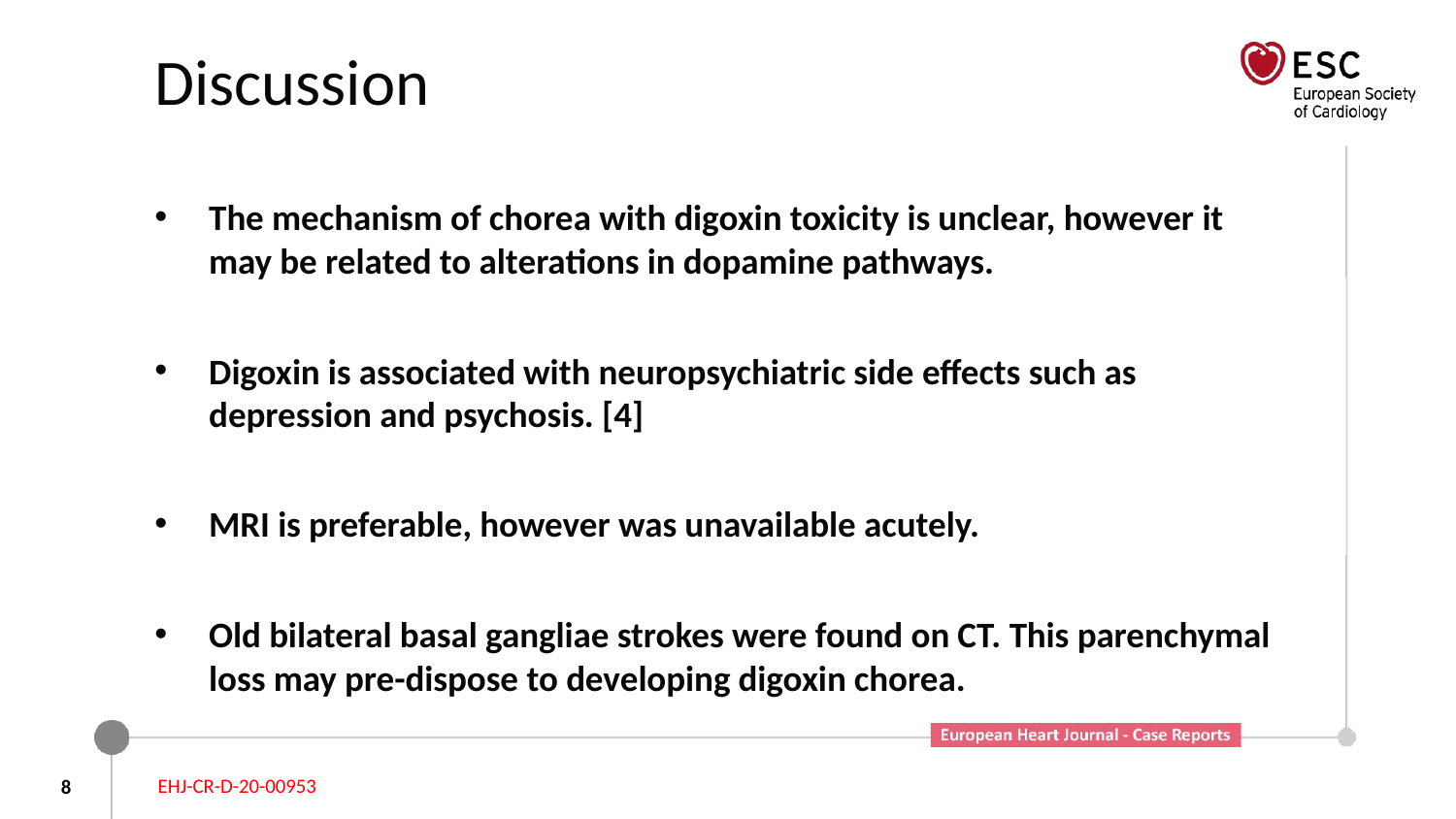

# Discussion
The mechanism of chorea with digoxin toxicity is unclear, however it may be related to alterations in dopamine pathways.
Digoxin is associated with neuropsychiatric side effects such as depression and psychosis. [4]
MRI is preferable, however was unavailable acutely.
Old bilateral basal gangliae strokes were found on CT. This parenchymal loss may pre-dispose to developing digoxin chorea.
8
EHJ-CR-D-20-00953

## Slide 9
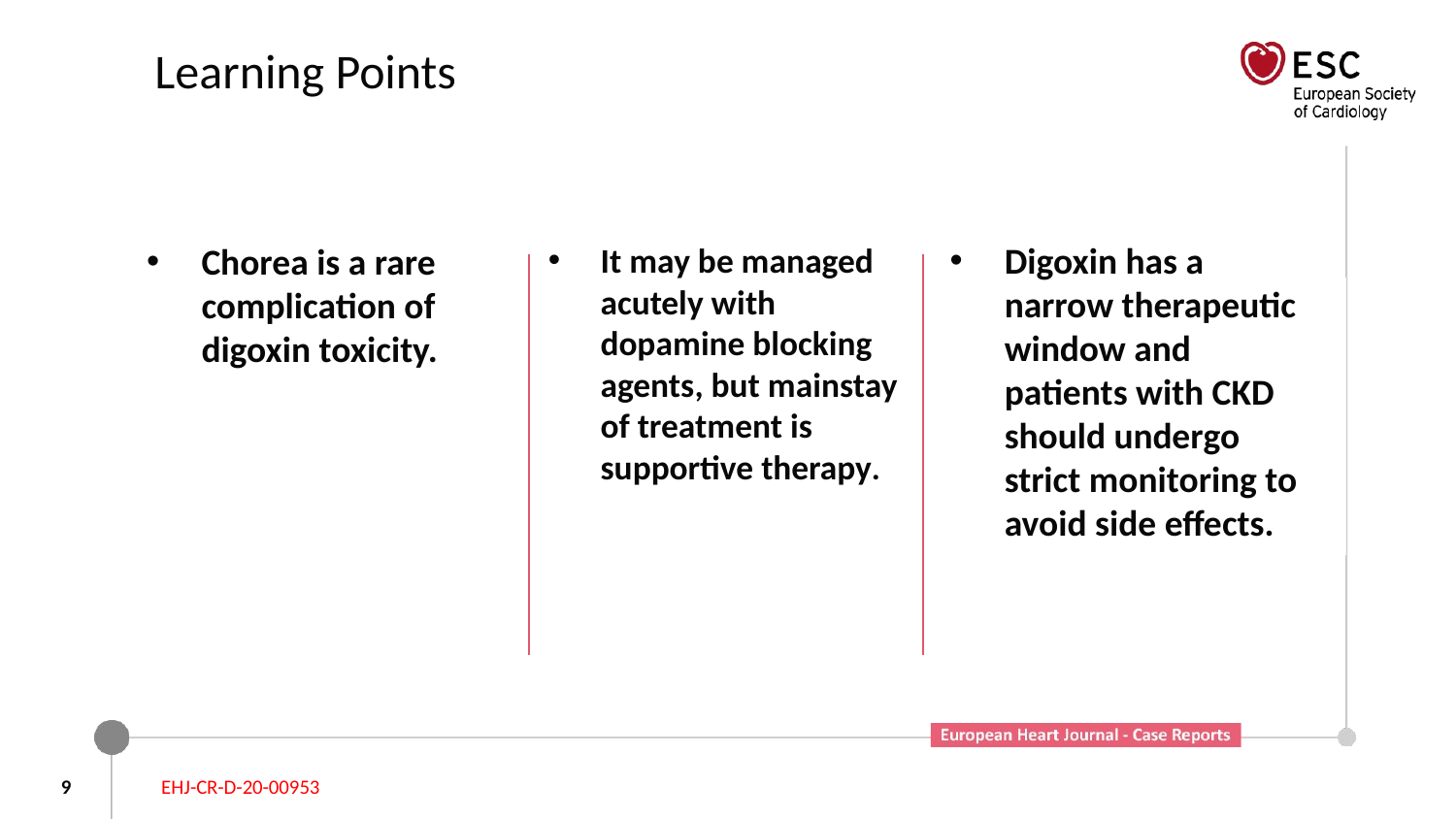

# Learning Points
Digoxin has a narrow therapeutic window and patients with CKD should undergo strict monitoring to avoid side effects.
Chorea is a rare complication of digoxin toxicity.
It may be managed acutely with dopamine blocking agents, but mainstay of treatment is supportive therapy.
EHJ-CR-D-20-00953
9

## Slide 10
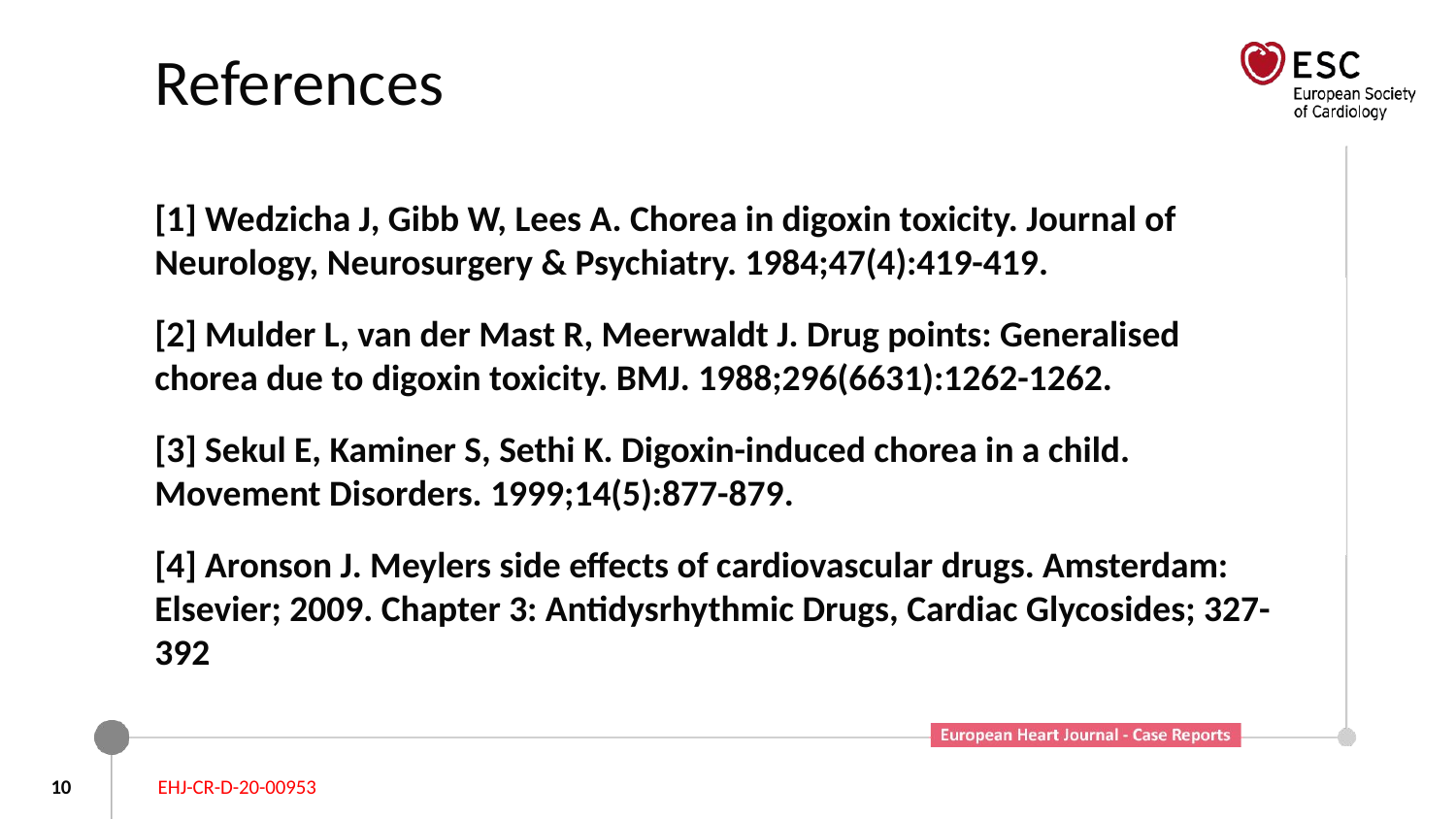

# References
[1] Wedzicha J, Gibb W, Lees A. Chorea in digoxin toxicity. Journal of Neurology, Neurosurgery & Psychiatry. 1984;47(4):419-419.
[2] Mulder L, van der Mast R, Meerwaldt J. Drug points: Generalised chorea due to digoxin toxicity. BMJ. 1988;296(6631):1262-1262.
[3] Sekul E, Kaminer S, Sethi K. Digoxin-induced chorea in a child. Movement Disorders. 1999;14(5):877-879.
[4] Aronson J. Meylers side effects of cardiovascular drugs. Amsterdam: Elsevier; 2009. Chapter 3: Antidysrhythmic Drugs, Cardiac Glycosides; 327-392
10
EHJ-CR-D-20-00953
